# Data on the extraction of benzoic, salicylic and sulfosalicylic acids from dilute solutions using PEG-based aqueous two-phase systems

**DOI:** 10.1016/j.dib.2019.105033

**Published:** 2019-12-19

**Authors:** Inna V. Zinov'eva, Yulia A. Zakhodyaeva, Andrey A. Voshkin

**Affiliations:** Kurnakov Institute of General and Inorganic Chemistry of the Russian Academy of Sciences, 31 Leninsky Prospect, Moscow, 119991, Russia

**Keywords:** Aqueous two-phase systems, Poly(ethylene glycol), Eco-friendly extraction, Distribution coefficient, Green chemistry, Aromatic acids

## Abstract

The use of green chemistry principles in the extraction of aromatic acids from dilute aqueous solutions has been considered. The extraction of a number of aromatic acids important for the food and pharmaceutical industries in heterogeneous systems based on poly(ethylene) glycol 1500 (PEG-1500) has been studied for the first time. This research presents a data of the quantitative characteristics of the extraction of benzoic, salicylic and sulfosalicylic acids using a PEG-1500 (15 wt %)/Na_2_SO_4_ (9 wt %) aqueous two-phase system under various conditions (temperature and рН). The effect of various phase-forming salts (Na_2_CO_3_, (NH_4_)_2_SO_4_, and (NH_4_)_2_HPO_4_) in a PEG-1500-based aqueous two-phase system on the extraction of aromatic acids has been found. For salicylic and sulfosalicylic acids, distribution coefficients when using (NH_4_)_2_HPO_4_ have been obtained that considerably exceed values for conventional water–organic solvent systems.

**Table:** Specifications Table

Subject	Chemical Engineering
Specific subject area	Separation and purification processes
Type of data	Tables, figures
How data were acquired	– Thermostatically controlled shaker (Enviro-Genie SI 12-02, Scientific Industries, Inc., USA) – Analytical balance (Ohaus Explorer, Zurich, Switzerland) – рН-meter (Starter 5000, OHAUS, USA) with a combined STMICRO5 RU glass electrode – High-performance liquid chromatograph isocratic Staier with spectrophotometric detector UVV 104.1М (Akvilon, Russian Federation) – Phenomenex Luna 3u C18(2) chromatographic column (150 × 3 mm) (USA), the mobile phase was composed of 0.5% phosphoric acid, 17% acetonitrile and 82.5% water – Centrifuge (CM-6MT, SIA ELMI, Latvia)
Data format	Raw and analyzed data
Parameters for data collection	All extraction experiments were carried out at a temperature of 25°С (excluding temperature dependences) and an atmospheric pressure of 100 kPa. The extraction of aromatic acids was carried out in centrifuge tubes (15 mL) with stirring for 15–20 minutes at a ratio of the volumes of phases of 1:1. Centrifugation was performed for 10 min at 2500 rpm.
Description of data collection	After extraction, volumes and pH values of the top and bottom phases were measured, the phases were separated, and the concentration of the acid in top and bottom phase was determined. The values of the distribution coefficient (D) and the degree of extraction (E,%) of organic acids were calculated.
Data source location	Kurnakov Institute of General and Inorganic Chemistry of the Russian Academy of Sciences, Moscow, Russia
Data accessibility	All raw data are available within this article.

**Table:** 

**Value of the Data** • An eco-friendly method for the extraction of aromatic acids from dilute aqueous solutions without the use of toxic, flammable and/or expensive organic extractants is proposed. • The data obtained demonstrate the high efficiency of ATPS for the extraction of aromatic acids (D > 8) in comparison with the currently used extractants. • Quantitative characteristics of the interfacial distribution for modeling the chemical-technological processes of separation and purification of aromatic acids from natural and man-made products are necessary. • The acquired data can be useful to the scientific community for the formation of new approaches based on the principles of “green” chemistry to solve the problems of extraction, separation and purification of liquid mixture components.

## 1 Data

**Fig. 1:**
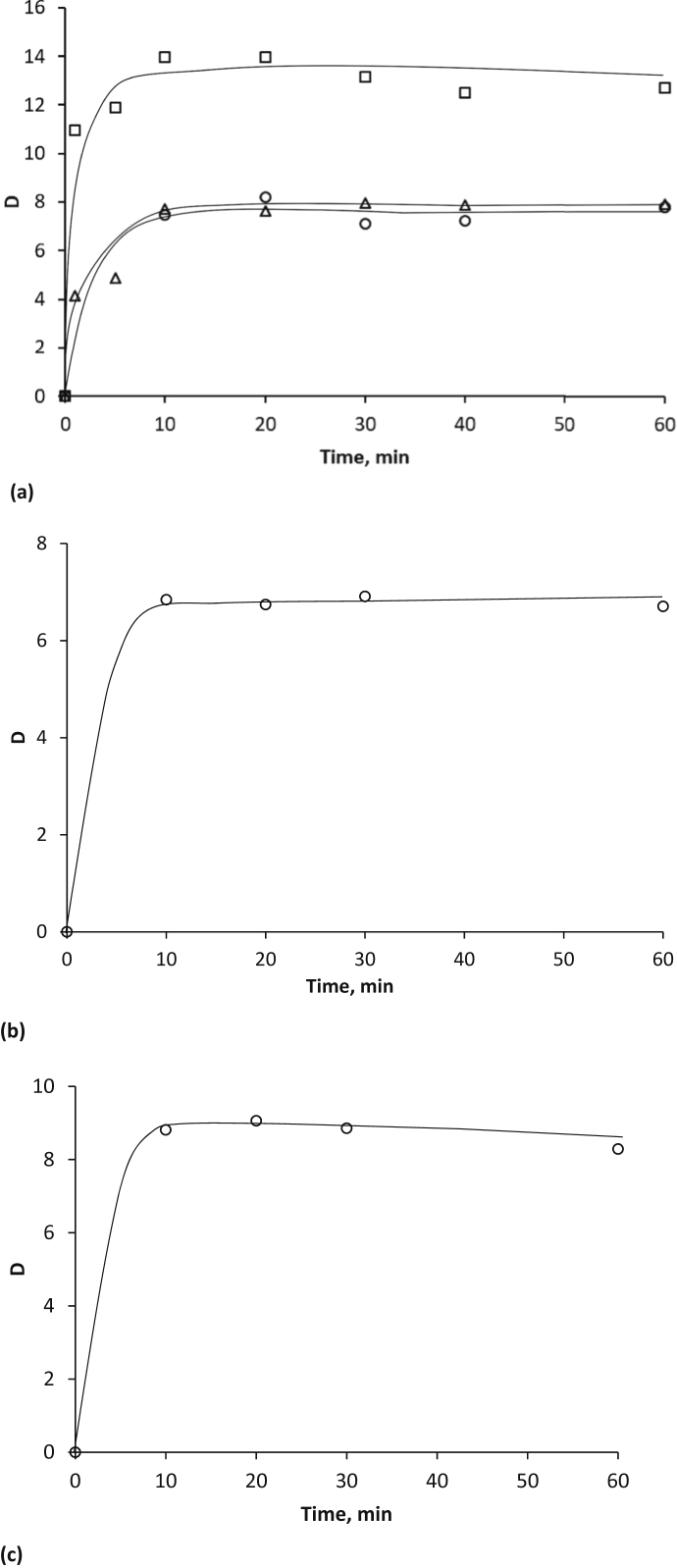
Dependence of distribution coefficients for aromatic acids on the time of phase contact in a) the PEG-1500 – Na_2_SO_4_ – H_2_O ATPS at 25°С: ○ – benzoic acid, □ – salicylic acid, and △ – sulfosalicylic acid, in b) the PEG-1500 – (NH_4_)_2_SO_4_ – H_2_O ATPS at 25°С, in c) the PEG-1500 – (NH_4_)_2_HPO_4_ – H_2_O ATPS at 25°С.

**Fig. 2:**
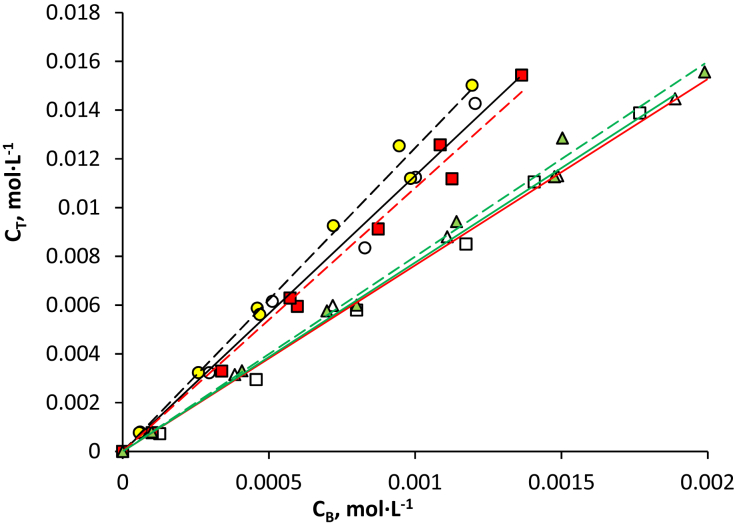
Isotherms of the extraction of aromatic acids using a PEG-1500 (15 wt %) – Na_2_SO_4_ (9 wt %) – H_2_O ATPS at 25 °C: ○ – salicylic acid, △ – benzoic acid, □ – sulfosalicylic (dotted line and filled markers – acid in individual solutions; solid line and blank markers – in mixture).

**Fig. 3:**
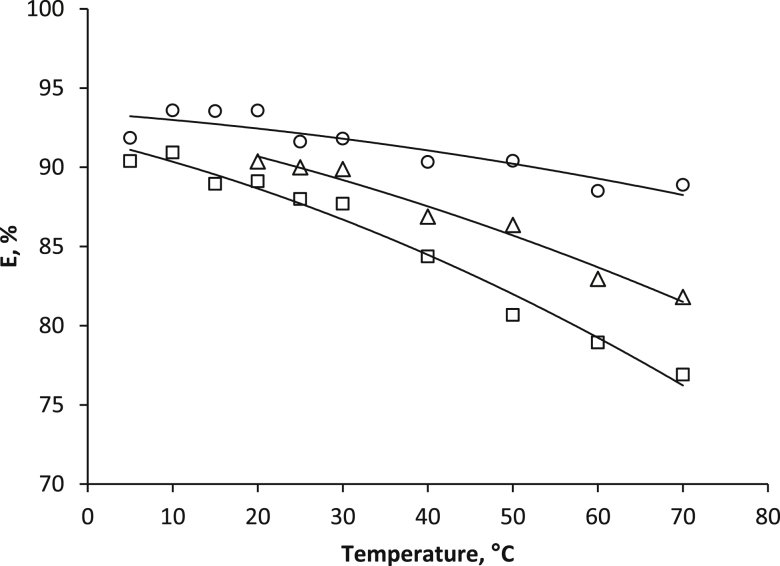
Degree of the recovery of aromatic acids as a function of temperature using the PEG-1500 (15 wt %) – Na_2_SO_4_ (9 wt %) – H_2_O system: ○ – salicylic acid, △ – benzoic acid, and □ – sulfosalicylic acid.

**Fig. 4:**
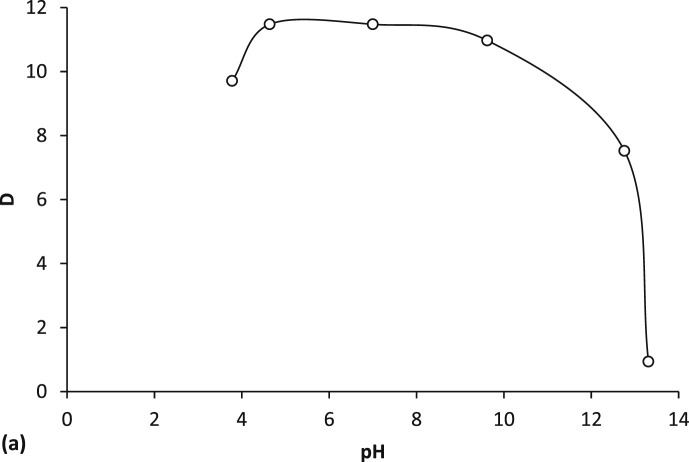

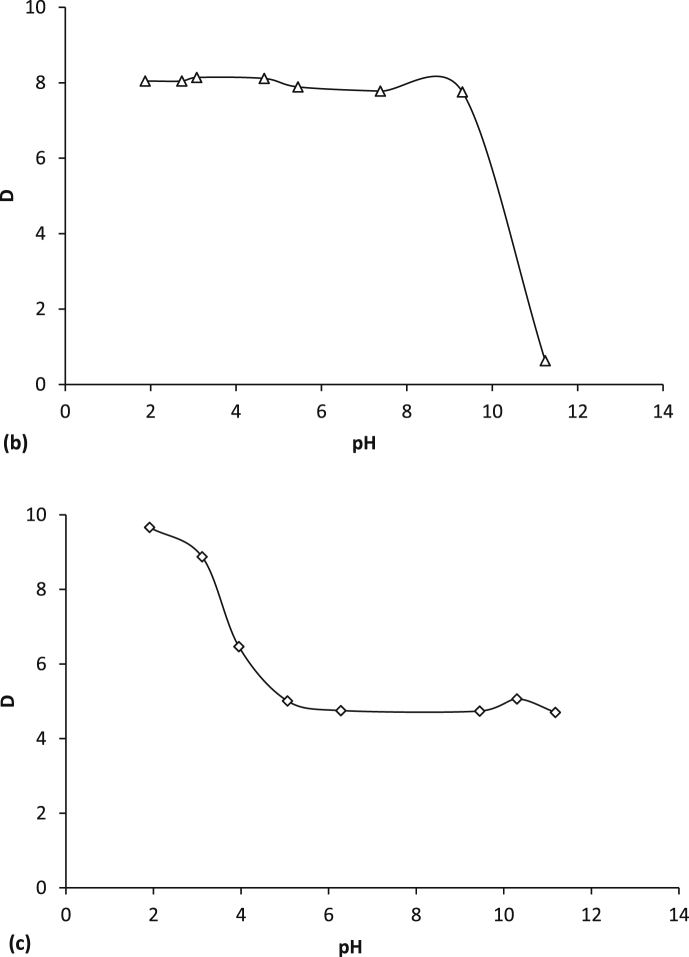
Dependence of distribution coefficients for aromatic acids on the рН value of the medium in the PEG-1500 (15 wt %) – Na_2_SO_4_ (9 wt %) – H_2_O ATPS: a – salicylic acid, b – sulfosalicylic acid, and c – benzoic acid.

Fig. 1 shows the kinetic dependences of the acids distribution coefficients in extraction systems based on PEG-1500 and inorganic salts Na_2_SO_4_, (NH_4_)_2_SO_4_, (NH_4_)_2_HPO_4_. Fig. 2 represents the isotherms of the aromatic acid extraction from individual solutions and their mixture. Fig. 3 and Table 1 show temperature effect on the extraction degree of aromatic carboxylic acids using PEG-1500 – Na_2_SO_4_ – H_2_O system. Fig. 4 shows the dependences of the distribution coefficients of the studied acids on pH values. Table 2 shows the effect of various inorganic salts on the distribution coefficient and the extraction degree of aromatic acids. Table 3 represents the comparative data on the distribution coefficients of aromatic acids obtained both in this study and in other works.

**Table 1:** Effect of temperature on the extraction of aromatic acids using a PEG-1500 – Na_2_SO_4_ – H_2_O ATPS.

Acid	Temperature (°С)	V_B_ (mL)	V_T_ (mL)	D (−)	E (%)
Benzoic acid	20	4.6	4.4	8.98	90.37
	25	4.4	4.6	9.40	89.99
	30	4.4	4.6	9.29	89.89
	40	4.2	4.8	7.57	86.88
	50	4.0	5.0	7.91	86.36
	60	3.6	5.4	7.30	82.96
	70	3.4	5.6	7.41	81.81
Salicylic acid	5	4.6	4.4	10.79	91.85
	10	5.0	4.0	11.69	93.59
	15	4.4	4.6	15.14	93.54
	20	4.4	4.6	15.24	93.58
	25	4.4	4.6	11.43	91.62
	30	4.4	4.6	11.72	91.81
	40	4.0	5.0	11.69	90.34
	50	3.7	5.3	13.51	90.41
	60	3.4	5.6	12.69	88.51
	70	3.2	5.8	14.51	88.89
Sulfosalicylic acid	5	4.6	4.4	9.00	90.39
	10	5.0	4.0	8.02	90.93
	15	4.4	4.6	8.42	88.95
	20	4.4	4.6	8.56	89.11
	25	4.4	4.6	8.70	88.00
	30	4.4	4.6	7.45	87.70
	40	4.0	5.0	6.75	84.37
	50	3.7	5.3	5.98	80.68
	60	3.4	5.6	6.17	78.94
	70	3.2	5.8	6.04	76.91

**Table 2:** Effect of a salt on the extraction of aromatic acids using ATPS with PEG-1500 (15 wt %).

Acid	No.	Salt	Salt content (%)	V_T_ (mL)	V_B_ (mL)	D (−)	E (%)
Benzoic acid	1	Na_2_SO_4_	9	4.4	4.6	8.80	89.00
	2	Na_2_CO_3_	9	3.6	5.4	7.20	82.70
	3	(NH_4_)_2_SO_4_	12	4.4	4.8	6.53	85.65
	4	(NH_4_)_2_HPO_4_	12	4.0	5.0	9.13	87.95
Salicylic acid	1	Na_2_SO_4_	9	4.4	4.6	12.90	88.00
	2	Na_2_CO_3_	9	3.6	5.4	18.95	92.49
	3	(NH_4_)_2_SO_4_	12	4.4	4.8	9.46	89.57
	4	(NH_4_)_2_HPO_4_	12	4.0	5.0	28.99	95.86
Sulfosalicylic acid	1	Na_2_SO_4_	9	4.4	4.6	8.70	88.00
	2	Na_2_CO_3_	9	3.6	5.4	7.73	87.63
	3	(NH_4_)_2_SO_4_	12	4.4	4.8	7.32	84.68
	4	(NH_4_)_2_HPO_4_	12	4.0	5.0	26.47	95.49

**Table 3:** Comparison of distribution coefficients for aromatic acids in various extraction systems.

Acid	Benzene	Toluene	Xylene	Hexane	Diethyl ether	Ethyl acetate	1-Hexanol	PEG-1500 – Na_2_SO_4_ – H_2_O
Benzoic acid	2.4–4.5 [3]	1.5 [3]	0.4–2.0 [3]	0.55	35.4	32.5	29.2	8.8
Salicylic acid	2.9–4.3 [3]	1.7–4.5 [3]	1.77	0.10	43.8	45	24	12.9
Sulfosalicylic acid	0.34	0.26	0.23	0	0	0	0	8.7

## 2 Experimental design, materials, and methods

### 2.1 Materials and reagents

Polyethylene glycol with a molecular weight of 1500 was purchased from Fluka (Shanghai, China). Na_2_SO_4_, Na_2_CO_3_, (NH_4_)_2_SO_4_, and (NH_4_)_2_HPO_4_ were used as phase-forming salts and were purchased from Fluka (Shanghai, China). Benzoic, salicylic and sulfosalicylic acids were used from Sigma-Aldrich (St. Louis, MO, USA) (99% purity). Acetonitrile (chromatographic grade) was purchased from Macron (Gliwice, Poland). Benzene, toluene, xylene, hexane, diethyl ether, ethyl acetate, 1-hexanol were obtained from Sigma-Aldrich (St. Louis, MO, USA). Sulphuric and phosphoric acids were purchased from Chimmed (Moscow, Russia). All solutions were prepared using distilled water purified in a UPVA-5 unit for the production of analytical grade water (Livam, Belgorod, Russia).

### 2.2 Extraction of aromatic acids using ATPS

To study extraction equilibria in aqueous two-phase systems, centrifugal tubes (15 mL) were used. Previously, the authors of [1,2] constructed phase diagrams for PEG-1500 – Na_2_SO_4_ (Na_2_CO_3_, (NH_4_)_2_SO_4_, (NH_4_)_2_HPO_4_) – H_2_O systems, using which the concentration of each component of an extraction system was chosen: PEG-1500 (15 wt %), Na_2_SO_4_ (9 wt %), Na_2_CO_3_ (9 wt %), (NH_4_)_2_SO_4_ (12 wt %), and (NH_4_)_2_HPO_4_ (12 wt %). To prepare the system, the necessary amounts of PEG-1500 and an inorganic salt were weighed using an analytical balance (Ohaus Explorer, Zurich, Switzerland) with an accuracy of ±0.0001 g, and water and aliquot of the working solution of an organic acid were then added. The initial concentration of benzoic, salicylic and sulfosalicylic acids in the systems was 0.01 mol/L. The extraction isotherms of aromatic acids were obtained by varying the initial acid concentration from 0 to 0.01 mol/L. After that, the system was agitated in a thermostatically controlled shaker with an accuracy of ±0.2 °C (Enviro-Genie SI 12-02, Scientific Industries, Inc., USA) for 15–20 minutes at a rotation speed of 30 rpm/min. After extraction, the extracted solution was centrifuged at 2500 rpm/min (CM-6MT, SIA ELMI, Latvia) for 10 min. The volumes of the top and bottom phases were then measured, the phases were separated, and the concentration of the acid in each phase was determined. 1 mL phase was sampled and diluted with water. The diluted solutions (100 μL) was injected into the high-performance liquid chromatography (HPLC) instrument for analysis. All the experiments were carried out three times and the average and standard deviation for data were calculated.

When building dependences between aromatic acids extraction and acidity, the required pH-value was reached by adding sulphuric acid which was controlled with an accuracy of ±0.001 by a рН-meter (Starter 5000, OHAUS, USA) with a combined STMICRO5 RU glass electrode calibrated against buffers with the рН-values 1.68, 4.01, 7.00, and 10.01 (at 25 °C).

### 2.3 Determination of the concentration of aromatic acids

The concentration of aromatic acids in the initial solution and in the top and bottom phases after extraction was determined by high-performance liquid chromatography. For this purpose, a Staier liquid chromatograph (Akvilon, Russian Federation) with a UVV 104.1М spectrophotometric detector was used. Chromatographic separation was achieved on an Phenomenex Luna 3u C18(2) column (150 × 3 mm). The mobile phase was composed of 0.5% phosphoric acid, 17% acetonitrile and 82.5% water. The flow rate was set at 0.3 mL/min and the column temperature was set at 25 °C. Spectrophotometric detection was conducted in the ultraviolet region at a wavelength of 230 nm using calibration curves.

To quantitatively describe and evaluate the efficiency of the extraction of organic acids, the distribution coefficient (D) and the degree of recovery (E) were used:

(1) $$D=\frac{C_{T}}{C_{B}}$$

(2) $$E=\frac{C_{T}\cdot V_{T}}{C_{in}\cdot V_{in}}$$

where С_B_, С_T_, and С_in_ are the concentrations of substances in the bottom phase, top phase, and initial solution, respectively, and V_T_ and V_in_ are the volume of the top phase and the initial volume of the system, respectively.

### 2.4 Comparison of the extraction of aromatic acids using various extraction systems

It is advisable to compare the extraction of aromatic acids in ATPS with data on the extraction of the studied objects in traditional extraction systems. Table 3 shows the distribution coefficients of aromatic acids obtained by us, as well as data from other studies. The extraction of aromatic acids with organic solvents was carried out in centrifuge tubes (15 mL) with stirring in a thermostatic shaker for 15–20 minutes at a ratio of the volumes of aqueous and organic phases of 1:1. After contacting the phases were centrifuged, separated, their volumes were measured and the concentration of aromatic acids HPLC in the aqueous phase was determined. The concentration of aromatic acids in the organic phase was determined by the difference between the concentrations in the initial solution and in the aqueous phase after extraction.
